# The New Health-Related Top-Level Domains Are Coming: Will Cureforcancer.health Go to the Highest Bidder?

**DOI:** 10.2196/jmir.3358

**Published:** 2014-03-05

**Authors:** Gunther Eysenbach

**Affiliations:** ^1^Techna InstituteCentre for Global eHealth InnovationUniversity Health Network and University of TorontoToronto, ONCanada

**Keywords:** top-level domains, global community health, health information sources, quality health information

## Abstract

In 2012, the Internet Corporation for Assigned Names and Numbers (ICANN) opened a new round of applications for generic top-level domain (gTLD) names, receiving 1930 applications, of which at least 18 were related to health (eg, “.doctor”, “.health”, “.med”). The entry of new, commercial players applying to create health-related names reopens the debate on the role of international organizations, governments, non-governmental organizations, and other stakeholders regarding the safeguards and policies needed to protect consumers.

## The New Health-Related Generic Top-Level Domains

In 2012, the Internet Corporation for Assigned Names and Numbers (ICANN) opened a new round of applications for generic top-level domain (gTLD) names, receiving 1930 applications, of which at least 18 were related to health (eg, “.doctor”, “.health”, “.med”; see Textbox 1 for the full list). The potential creation of new health-related names by strictly commercial players reopens the debate on the role of international organizations, governments, non-governmental organizations, and other stakeholders regarding the safeguards and policies needed to protect consumers [1].

As the paper by Mackey and colleagues in this issue of Journal of Medical Internet Research (JMIR) shows [1], the global health community is in the process of losing an important battle: the sell-off of health-related gTLDs to the highest bidders, forfeiting a potential asset and unique opportunity to promote health. Despite multiple objections and concerns raised by different stakeholders, including the World Health Organization (WHO) [2], ICANN appears to forge ahead with its current plans to assign the administration of health-related gTLDs to operators whose business models are not necessarily aligned with public health objectives and without sufficient safeguards that are based on a community consensus. In fact, it appears that other top-level domain names like .bingo or .wtf receive more consumer protection and regulation than health-related top-level domains. For example, ICANN created additional safeguards for domains like .wtf or .sucks (asking top-level domain operators to define and implement policies against cyberbullying), but policies that ensure certain minimum standards for health information are lacking. ICANN has put generic safeguards in place for areas that are “highly regulated” but certain health-related domains like .health are on the auctioning block with only 3 minimal and generic safeguards such as removal of illegal content.

**Some proposed new health-related top-level domain names (number of applications in brackets, if more than one).**

- .health (4, one of which is withdrawn)
- .med (4, one of which is withdrawn)
- .doctor (3)
- .fit (2)
- .healthy (Chinese variant)
- .healthcare
- .medical
- .hospital
- .pharmacy
- .skin
- .surgery
- .heart (withdrawn)
- .hiv
- .clinic
- .dental
- .dentist
- .cialis (withdrawn)
- .fitness

**Figure 1:**
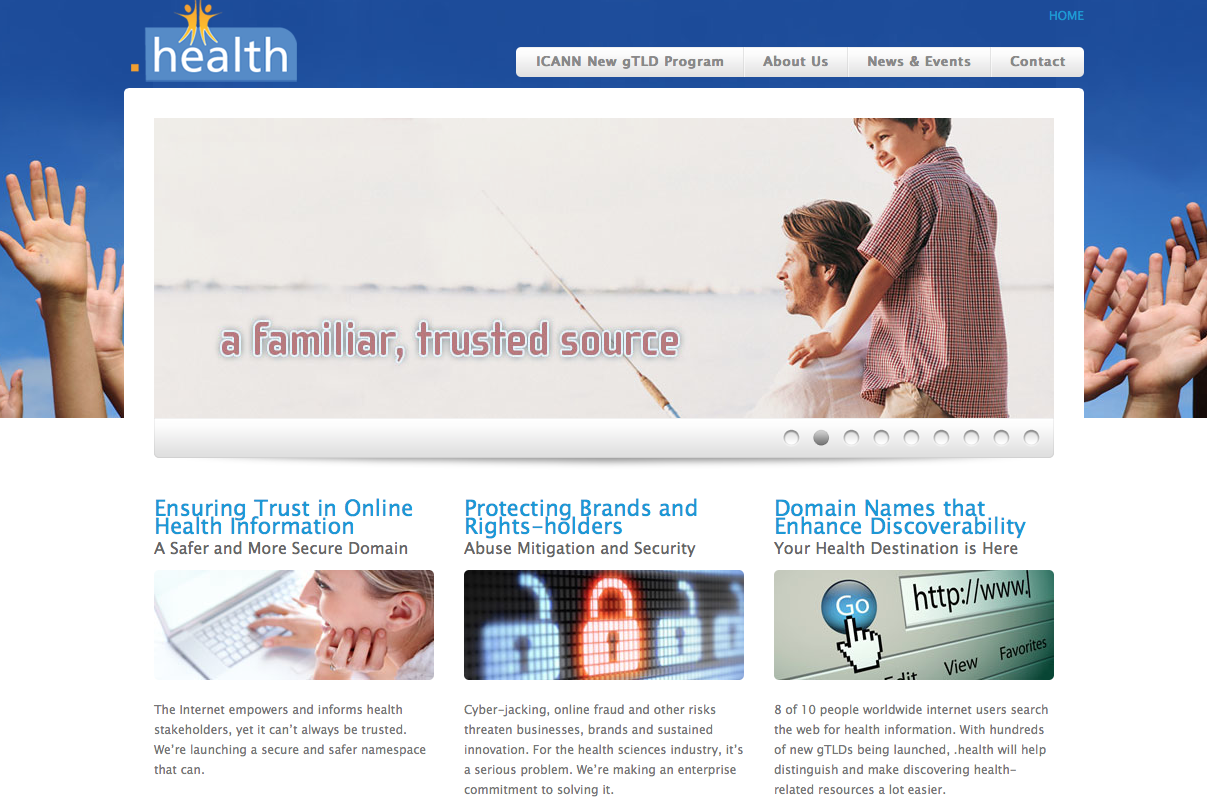
Screenshot of DotHealth LLC.

**Figure 2:**
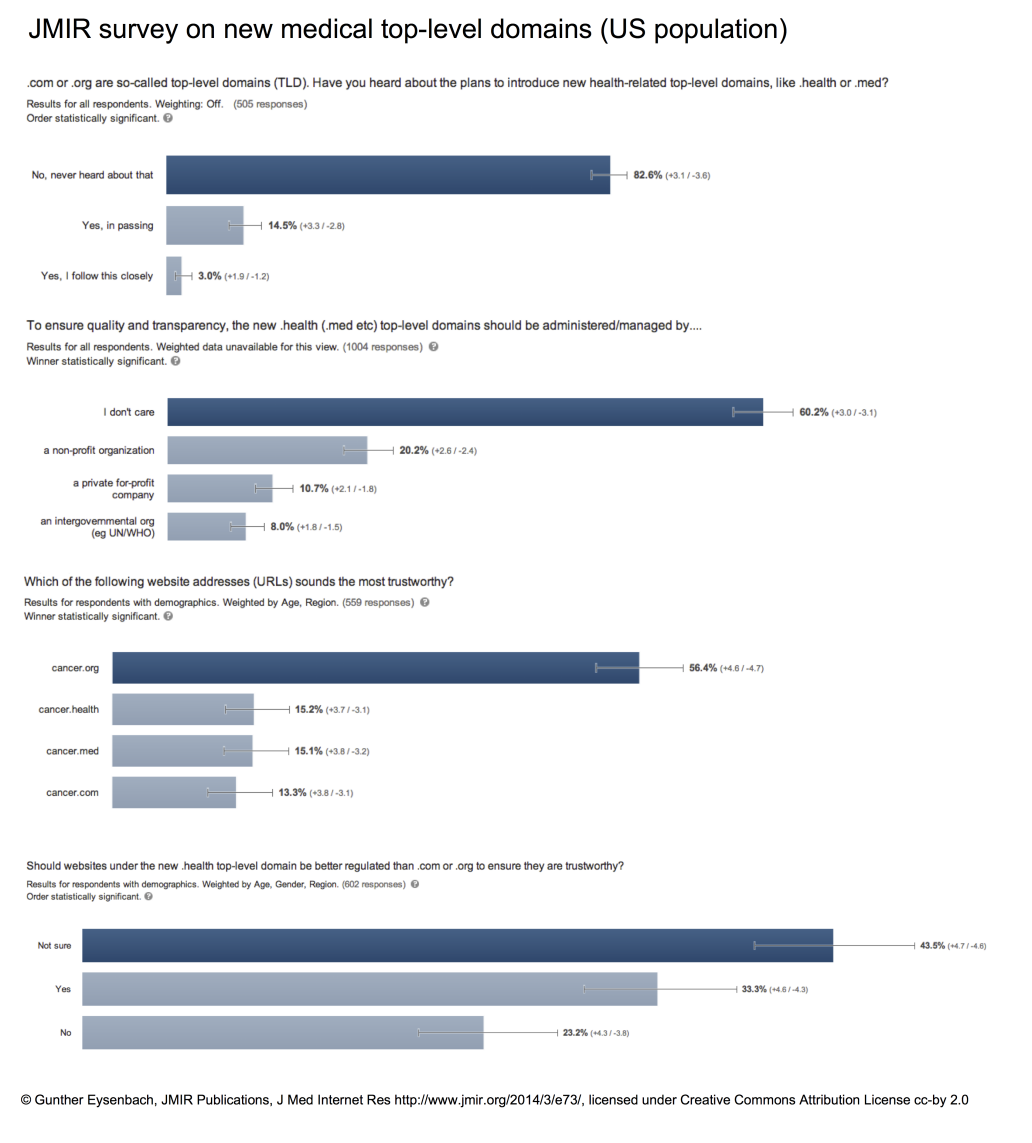
JMIR polling results among the US population regarding health-related top-level domains.

Some potential operators of health-related gTLDs already promote their namespace as “trusted” (see Figure 1), and according to a JMIR poll, many users intuitively trust a health-related domain more than a .com domain (see Figure 2 and below). Hence, ICANN and gTLD operators have an ethical responsibility to implement appropriate safeguards and industry standards which go beyond the removal of illegal content, and to involve experts or organizations which have experience in assessing health information and in public health in the design of their processes and in their ongoing operations. Some gTLD applicants made a superficial attempt to balance commercial interests with public health objectives, and walk a difficult line between promising a “trustworthy” environment while trying to avoid expensive, time-consuming and potentially subjective examination of potential domain owners’ source credibility. In an interview with JMIR, Andy Weissberg, CEO of DotHealth LLC, who is one of the remaining 3 contenders for the .health gTLD, explains that under their proposal, “harmful and illegal information will be removed” (as is expected for all gTLDs), but also states that “attempting to keep information off the .health gTLD in the name of ‘quality’ is a dangerous precedent that amounts to potential censorship of free speech at worst and favoritism at best”. This perspective fails to acknowledge that quality assurance is not so much about censorship and “keeping information off” the Internet, but perhaps more about soliciting and providing *additional* information on prospective domain owners, for example conflicts of interest in the form of additional fields in WHOIS directories or standardized metadata [3,4]. No single body (let alone the domain registrar) should determine what is “correct” health information. It can not be the goal to “censor” content or the messages on .health websites. It will always remain up to the website owners to ensure “*message credibility*”, and will always remain the responsibility of users to learn how to distinguish quality sites (“caveat lector”) [5]. A gTLD can, if anything, only be a very indirect “quality label” for content, not least because when prospective applicants apply for the second level domain name, there is not necessarily any content to evaluate at that time, and withdrawing the address after content has been created would be a rather drastic and litigious measure unless there is blatantly illegal or harmful information. Thus, this debate should be less about *content* quality, rather, it should be about *source* quality. If the goal is to make the health-related domains a trusted space, then principles of source credibility must be implemented, and *transparency* should be the guiding principle to allow consumers to judge the *expertise* and *trustworthiness* of the source [4]. For dot-coms and other domains, it may be acceptable for the site owner to hide their identity and biases, but in health it simply is not [3-11]. It must be transparent at all times who the site owner is and what his potential biases are, and what the mechanisms are to maintain privacy, security, and confidentiality of medical and personal data, so that users can make their own judgments about the health information, products, or services provided by the site. These universal principles have been implemented in various ethical codes and health information quality initiatives on the Internet for over a decade [3-11], and should be operationalized at the registrar if they claim to operate a “trusted” namespace .

Source credibility can be achieved in two ways: (1) an “upstream” evaluation by the registrar requiring certain credentials or criteria for the prospective second-level domain owner (for example ISO certification, professional licenses, educational degrees or other credentials), and/or (2) by a workflow where registrars take additional steps to ensure that site owners declare their financial interests and disclose their credentials and privacy protection mechanisms, making this information transparent (and machine-readable) so that users can judge for themselves if the source is trustworthy, and software can assist users in finding relevant and trustworthy information for their specific purpose. One approach to achieve this is through a simple but mandatory questionnaire to site owners when they apply for a second-level domain name. This metainformation should be viewable and searchable by consumers, and perhaps be mandated to be provided on the sites themselves as machine-processable meta-tags (metadata), which would make it possible for the site owner to change the metadata, or to have different metadata for different sections of the site, as suggested in the MedCERTAIN/MedCIRCLE projects [3,4,9]. In addition, this would allow domain registrars to automatically monitor the presence of disclosure statements, and allow search engines to further improve and filter search results.

Such considerations are currently not included in any of the applicants’ documents. While DotHealth LLC is planning a “Request for Information” (RFI) process for selected second-level domain names which include, for example, disease names [12], it is unclear to what degree the information obtained will be publicly accessible, useful, or even machine-processable. Moreover, the proposed RFI process would only apply to a limited subset of second-level domain names under .health. Weissberg also stressed in an interview with JMIR that it would be “unacceptable if we were to in some way ‘discriminate’ the allocation of a reserved name or any .health name based on a prospective registrant's source credibility, financial interests or ‘prescriptive’ approaches to treating a disease/condition as being more favorable to over another registrant's non-commercial status or methods of treating a disease or condition.” In other words, if a pharmaceutical company wanted to own mental.health to promote its psychotropic medications, it could do that, even if it were biased against non-pharmaceutical treatments such as psychotherapy, and there is nothing wrong with that, unless the consumer is not aware of the fact that the information offered is biased due to commercial interests. The RFI process is a step in the right direction, but the information obtained by the registrar should be public, and it also appears that questions critical for full transparency (eg, financial interests) are not asked or disclosed. If this level of transparency were present, then under the proposed framework above, consumers would at least still be able to identify the source and its potential biases. Apart from principles of transparency, there are other essential criteria for health information sources, such as privacy and confidentiality. 

Are the proposed public interest commitments of the current applicants for health-related domains enough? Many in the global health community do not think so. The WHO received a mandate at the 9^th^ plenary meeting of the World Health Assembly on May 27^th^, 2013, to “convey to the appropriate bodies, including the ICANN GAC and ICANN constituencies, the need for health-related global top-level domain names in all languages, including “.health”, to be consistent with global public health objectives”[13]. It is currently unclear if the proposed public interest commitments of applicants are sufficient to meet this ambitious goal. No less than a dozen organizations have expressed reservations or objections, including the Cochrane Collaboration and the International Medical Informatics Association (IMIA) [14]. These objections were dismissed by a legal expert ruling on the objections, essentially implying that an organization that has “medical informatics” in its name is no more authorized to speak on behalf of the global health community than UFO enthusiasts speaking out against .astrophysics [14]. If concerns expressed by WHO and by international professional medical societies are not deemed representative for the health community, then who is authorized to speak for global health? And where are the consumers and patients in this debate?

## Public Opinion: A Poll by JMIR

Where does the public stand on this issue and where are the voices of patient and consumer organizations? As the debate has not entered mainstream media, there has not been much (if any) debate. According to a poll conducted by JMIR Publications in February 2014 among Internet users from the US, over 80% of consumers have not heard about the new health-related gTLDs, and most are indifferent about the question who should administer health-related gTLDs (60.2% said they “don’t care”), but among those who cared, a clear majority is against the idea that they should be managed by a private for-profit company (only 10.7% were comfortable with this idea), while most favored a non-profit organization to be in charge (20.2%) (Figure 2), and an additional 8.0% want an international organization (WHO) in charge.

Another poll conducted by JMIR Publications reveals that 43% of respondents are unsure if .health should be better regulated than .com or .org domains, but among those who have an opinion on this question, a slight majority thinks that this should be the case, with 33.3% of all respondents favoring more regulation and only 23.2% saying that this should not be the case (Figure 2).

A fourth JMIR poll confirms that gTLDs enjoy different levels of “credibility” among users (Figure 2), with the .org domain being the most trusted gTLD. This is consistent with earlier research published in this journal [15], but surprisingly, the yet-to-be-created and largely unknown gTLDs .med and .health enjoy at least the same, if not higher credibility than .com, with no statistically significant differences between them (Figure 2).

### Conclusions

Health related information and data occupy a crucial and unique status on the Internet. A domain name is associated with a site’s brand, origin, content or quality. The sites that fall under .health are likely to be considered as the ultimate online source of information and advice on health, in particular by populations with less ehealth literacy. The marketing of .health as a trusted name, when it is not warranted, creates the likelihood of material detriment. The .health gTLD has been the 8th most contested name of the over 1900 gTLDs proposed—for good reason. It is time for ICANN to listen to the health community. The issue of how to define “quality health information” has been subject of much research and debate over the past decade, and contrary to what some applicants have implied, there is more consensus than disagreement over the criteria that should be taken into account when assessing health information quality and credibility [3-11]. What is lacking (and must be discussed in the context of gTLDs) is a consensus on *how these standards can and should be operationalized in the context of a domain registry*. We call for a delay in issuing the .health gTLD and other health-related gTLDs until adequate safeguards based on community consensus are in place.

However, given how readily the ICANN committees and their legal experts have brushed aside concerns from the health community, the most likely outcome is that a flood of new health-related gTLDs will enter the market late 2014 or 2015, marketing their gTLD as trustworthy to consumers. In this case, we urge any successful gTLD registries to seek collaboration with the health community and to reach out to individuals and organizations (including patient organizations) who have spent decades in conducting research on what quality health information means and how source credibility and technical criteria can be monitored. In the absence of that, perhaps it is time for the trusted players in the health community to apply for gTLD programs in a forthcoming round (for example, .who, .medcertain) that implement some of the suggestions related to transparency above, or to even go further by forming a large collaborative non-profit consortium which awards domain names under a new gTLDs based on the second-level domain applicants proposals and expertise, as opposed to their ability to pay. For consumers and patients, the adage “caveat lector” [5] remains crucial, and extends to having to learn about the different health-related top-level domains and the different levels of protection and “trustworthiness” they offer.

## Abbreviations

gTLD: generic top-level domain
ICANN: Internet Corporation for Assigned Names and Numbers
JMIR: Journal of Medical Internet Research
RFI: Request for Information
WHO: World Health Organization
